# Genetic *k*-Means Clustering Approach for Mapping Human Vulnerability to Chemical Hazards in the Industrialized City: A Case Study of Shanghai, China

**DOI:** 10.3390/ijerph10062578

**Published:** 2013-06-20

**Authors:** Weifang Shi, Weihua Zeng

**Affiliations:** State Key Laboratory of Water Environment Simulation, School of Environment, Beijing Normal University, Beijing 100875, China; E-Mail: swf06@mail.bnu.edu.cn

**Keywords:** vulnerability mapping, *k*-means clustering, genetic algorithm, silhouette coefficient, industrialized city

## Abstract

Reducing human vulnerability to chemical hazards in the industrialized city is a matter of great urgency. Vulnerability mapping is an alternative approach for providing vulnerability-reducing interventions in a region. This study presents a method for mapping human vulnerability to chemical hazards by using clustering analysis for effective vulnerability reduction. Taking the city of Shanghai as the study area, we measure human exposure to chemical hazards by using the proximity model with additionally considering the toxicity of hazardous substances, and capture the sensitivity and coping capacity with corresponding indicators. We perform an improved *k*-means clustering approach on the basis of genetic algorithm by using a 500 m × 500 m geographical grid as basic spatial unit. The sum of squared errors and silhouette coefficient are combined to measure the quality of clustering and to determine the optimal clustering number. Clustering result reveals a set of six typical human vulnerability patterns that show distinct vulnerability dimension combinations. The vulnerability mapping of the study area reflects cluster-specific vulnerability characteristics and their spatial distribution. Finally, we suggest specific points that can provide new insights in rationally allocating the limited funds for the vulnerability reduction of each cluster.

## 1. Introduction

Modern industrialization, specifically industries using or producing harmful substances and hazardous chemicals, pose considerable risks to human safety and the environment worldwide [1]. Chemical industry plants in industrialized cities are often established in dense urban areas; thus, a sizeable number of people are placed at risk. The health and safety of people in these cities have aroused increasing public concern. Thus, how to effectively reduce the risks of chemical hazards in an industrialized city has been a crucial issue. Corresponding technological support is necessary to achieve this objective.

Vulnerability analysis is a powerful analytical tool for describing the states of susceptibility to harm, powerlessness, and marginality of both physical and social systems, as well as for guiding normative analysis of actions to enhance well-being through risk reduction [2,3]. This information can contribute to understanding better the community and its environmental needs and to enabling the prioritization of limited resources in response to hazards [4,5,6]. Thus, vulnerability analysis is increasingly being regarded as a key step towards effective risk reduction and as the promotion of disaster resilience [7]. The term “vulnerability” is now a central concept in a variety of research contexts and has been used in many different ways [8,9,10,11,12].

Generally, vulnerability conveys the idea of susceptibility to damage or harm [13], characterizing how impacts are manifested and responded to the harm experienced or to be experienced from such impacts [14]. Literature on sustainability science denotes that vulnerability is the degree to which a system, a subsystem, or a system component is likely to experience harm due to exposure to a hazard, either a perturbation of stress/stressor [15]. This definition implies that vulnerability is the potential result of the interaction between a system and its environment.

Many studies have frequently characterized vulnerability as the interrelation of exposure of a system, sensitivity to stress, and the capacity to absorb or cope with the effects of these stressors as the potential of the system to decrease the impact of the hazard [2,15,16,17]. Exposure is defined as the degree, duration and/or extent in which a system is in contact with or subject to perturbation [2,18]. Sensitivity reflects the degree of a system affected with respect to the impact of the hazard. Coping capacity is the ability of a system to cope with or adapt to hazard stress.

Thus, we describe that vulnerability is the degree and extent of the potential damage from normal states or functions as a system or a community responds to the exposure imposed by hazards or stresses. Based on this conceptualization, the human vulnerability to chemical hazards in an industrialized city is understood to be shaped by the sensitivity and coping capacity of socio-ecological system and the exposure of system to chemical hazards generated by the release of chemicals from industry plants. This depiction allows the identification of an industrialized city population that is potentially affected by chemical hazard.

The purpose of vulnerability analysis is to reduce the negative effects of hazard, improve adaptation planning, unveil social injustices, and provide impetus for mitigation disasters, rather than produce a score or rating of the current or future vulnerability of a community [19]. To achieve this intention in a region, vulnerability analysis results require applying in spatial planning. Consequently, vulnerability mapping is an alternative approach as it enables the representation of vulnerability analysis through spatial rendering of geographically heterogeneous determinants of vulnerability and their interactions [20,21,22].

In this paper, we seek to provide an approach for mapping human vulnerability to chemical hazards via clustering analysis for effective vulnerability reduction. Conventional vulnerability studies [23,24,25,26] usually merge vulnerability dimensions into one final value, such that the individual dimensions are often not discernable in assessment results. Thus, the contribution of one dimension to the final value can be substituted and compensated for by other dimensions. The heterogeneity of vulnerability in an area is seldom considered. Areas exhibiting similar assessment results may have distinct vulnerability dimensions in space. As a result, vulnerability-reducing interventions cannot be targeted and prioritized to mitigate potential losses in an effective manner.

By contrast, clustering analysis keeps specific vulnerability dimensions transparent. The areas with similar characteristics in terms of vulnerability are categorized into the same cluster; that is, areas from other clusters have different characteristics in terms of vulnerability. This categorization is beneficial to the application of appropriate vulnerability-reducing measures in specific areas. In this study, *k-*means clustering is used because of the suitability of the method in clustering large data sets. However, *k-*means clustering requires the definition of a number of clusters in advance. Moreover, the clustering result is sensitive to the selection of initial cluster centers, which may result in the convergence of the algorithm to the local optima. To avoid these drawbacks, the genetic algorithm (GA) is used to determine good initial centers to attain a globally optimal partition. The sum of squared errors (SSE) and the silhouette coefficient are combined to measure the quality of clustering and to determine the optimal clustering number (*k*_opt_). We categorize the area according to the clustering result with *k*_opt_ as the clustering number. Finally, human vulnerability is mapped by using the geographic information system (GIS). 

To illustrate the advantage of the proposed approach, a case study is introduced in Shanghai, China. Nonparametric one-way analysis of variance (ANOVA) is performed to further analyze the validity of the clustering results by using Kruskal-Wallis test. Since clustering does not generate vulnerability ranking, we conduct an additional evaluation of the clusters on the basis of information entropy theory.

## 2. Materials and Methods

### 2.1. Study Area

**Figure 1:**
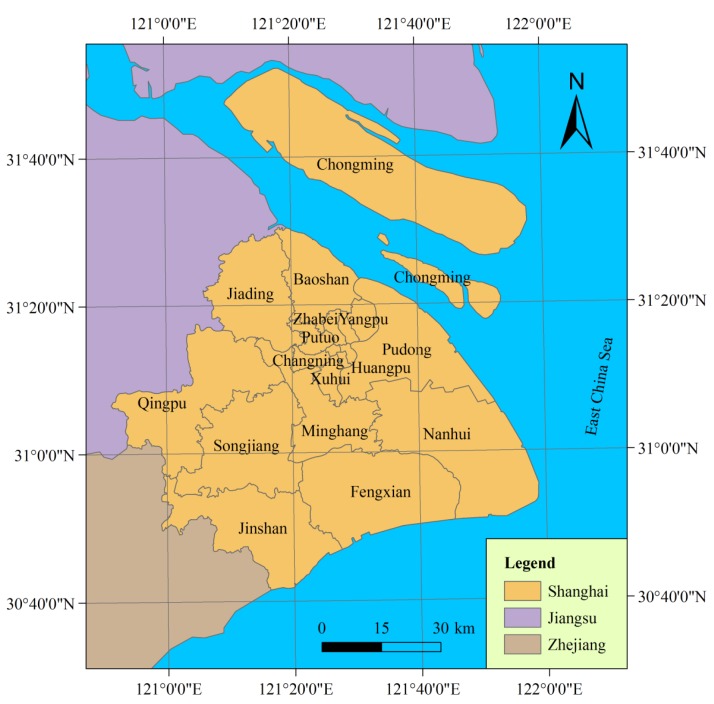
Location of the study area.

Shanghai, which has a total population of over 23 million as of 2010, is the largest city by population in the People’s Republic of China. It sits at the mouth of the Yangtze River in the middle of the Chinese coast, between the latitude 30°41′33.07″ to 31°52′4.25″ North and between the longitude 120°51'12.03″ to 121°58′49.17″ East, covering a total area of approximately 6,340.5 km^2^ (Figure 1). The city borders to Jiangsu and Zhejiang Provinces to the west and is bounded to the east by the East China Sea. Occupying part of the alluvial plain of the Yangtze River Delta, Shanghai lies generally on a flat and low-lying land, with the exception of some hills in its western regions. Its altitude varies between 3 to 5 m above mean sea level and increases from east to west.

Shanghai is an important economic and financial center in China. The great economy achievement of the city benefited from the amazingly rapid industrial development, with the automobile, electronic and communication equipment, petrochemical, steel product, equipment assembly, and biomedicine industries being promoted as the six pillar-industries. The industries in the city are still given considerable attention, and industrial production continues to grow rapidly. Though generating much economic profit for the city, these industries affect the environment and pose risks to citizens.

### 2.2. Quantitative Dimension of Vulnerability

In this paper, human vulnerability to chemical hazards involves exposure, which is the degree of a human community in contact with chemicals, sensitivity, which is the degree of a receptor affected when exposed to chemical hazards, and coping ability, which is the ability of a receptor to resist or recover from the damage associated with exposure to chemical hazards. Exposure and sensitivity place targets in potential dangers, whereas lack of coping ability reflects the inability of targets to respond to hazards.

#### 2.2.1. Exposure

Some proximity models are available for measuring human exposure. The simple nature of proximity models allows for their wide use in exposure assessment studies with few data requirements. These models are based on the assumption that exposure at locations nearer to emission sources are higher compared with locations further from the source. However, some parameters, such as emission rate and physicochemical characteristics of the emitted substances, are not considered in these models.

Zou *et al.* [27] developed an emission weighted proximity model (EWPM) to calculate the relative individual exposure from the traditional proximity model. EWPM considers the emission rate and emission time of each source. The formula for calculating exposure values on the basis of EWPM is as follows:

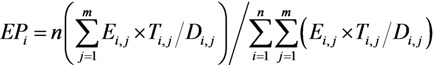
(1)
where *E_i_*_,*j*_ and *T_i_*_,*j*_ are the emission rate and emission time of the *j*th emission source, which the *i*th receptor is exposed to, respectively; *D_i_*_,*j*_ is the distance of the *i*th receptor to the *j*th emission source; *m* is the number of emission sources; *n* is the number of receptors.

However, this model is only suitable when all sources emit the same hazardous substance. Sources in a region may emit different hazardous substances. A source may even emit more than one type of hazardous substance. Therefore, we modify Equation (1) by considering the toxicity of each hazardous substance, as expressed in the following equation:

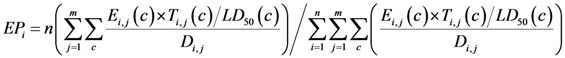
(2)
where *c* represents a dangerous chemical; *LD*_50_(*c*) is the median lethal dose of chemical *c* (g/kg), which is the dose, given all at once, causing the death of half the members of a group of test animals [28,29,30]. The *LD*_50_ is frequently used as a general indicator of the acute toxicity of a substance [31,32], and we use this figure to determine toxicity to humans. For the study area, the main corporations or plants concerning hazardous chemicals are taken into account to calculate the levels of exposure, which directly reflect the potential hazards that humans are exposed to with respect to the vulnerability.

#### 2.2.2. Sensitivity

Population density, which provides information on spatial concentration and distribution of people, is used to indicate the sensitivity of the study area in this work. Generally, highly dense areas with high population concentration show higher vulnerability to hazards compared with lowly dense areas, for hazards occurring in areas with denser population will result in greater harm than in less dense areas. For instance, a large, severe leakage of hydrogen sulfide that passes through an open field presents little danger. By contrast, a relatively weak leakage of the same substance can pose significant risks to human life in densely populated areas. In addition, widely available open spaces in lowly dense areas can function as refuge bases and as disaster recovery bases in times of emergency. In short, the higher the population density and the more compact the area is, the heavier the loss a community will suffer when exposed to hazards. Therefore, population density is of great importance in indicating the sensitivity of human vulnerability in an area, directly reflecting the degree of damage when exposed to hazards.

#### 2.2.3. Coping Capacity

By considering the coping capacity, we screen indices on income, medical service supply, and access to social resources, such as hospitals. The gross domestic product (GDP) per capita represents the general income of an area. High levels of this feature usually result in the construction of high-quality infrastructure, installation and maintenance of early warning systems, modern civil protection, and the compensation of costs for reconstruction in disaster-struck areas. With these complete infrastructures and high-level emergency management, human vulnerability will be reduced. Thus, high value of GDP per capita will result in low vulnerability.

Hospital beds per 10,000 population can represent the medical treatment level of an area. The value of this indicator directly reflects the abilities of an area in rescuing and providing health care for the people. A high value of this indicator denotes that the medical treatment level of an area is high, which leads to a high level of health care and rescue. As a result, the damage of human by chemicals will be relieved, and the vulnerability will be mitigated.

Access to social resources is critical in resisting chemical hazards. For instance, in a community close to evacuation routes and hospitals, social resources are potentially facilitated by and are correlated to the distance of the community to the nearest main road. Therefore, this study uses the distance to the nearest main road to indicate the access of an area to social resources; that is, the longer the distance of an area to the nearest main road, the lower the level of accessibility of an area for evacuation and rescue during emergencies. Thus, people in those areas with long distance to the nearest main road will be in high risk of damaged, *i.e.*, with high vulnerability.

The three aforementioned indicators can reflect the coping capacity of an area. These indicators are related to the capacity to cope with, resist, and respond to the effects when exposed to chemical hazards, significantly affecting human vulnerability. The GDP per capita is an indirect indicator, via affecting available social resources in contact with vulnerability. Hospital beds per 10,000 population and distance to the nearest main road are direct indicators with respect to vulnerability. The former reflect the rescue and health care providing abilities; the latter reflect the access to social resources and evacuation abilities. In addition, the negative relationship of GDP per capita and Hospital beds per 10,000 population with vulnerability is observed; that is, as the GDP per capita or Hospital beds per 10,000 population is increasing, the vulnerability is decreasing.

### 2.3. Genetic k-Means Clustering and Vulnerability Mapping

To obtain a precise vulnerability distribution in space, a 500 m × 500 m geographical grid is used as the basic spatial unit for mapping the vulnerability of Shanghai. Each grid cell is estimated by using the values of the five previously described indicators on human vulnerability. The indicators are then normalized as follows:

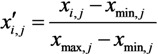
(3)

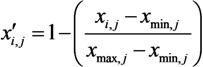
(4)
where *x′_i,j_* and *x_i,j_* respectively represent the normalized and original values of the *j*th indicator of the *i*th grid, and *x*_min__, *j*_ and *x*_max__, *j*_ respectively represent the minimum and maximum values for the *j*th indicator of all grid cells. Equation (3) is applied to the indicators of exposure, population density, and distance to the nearest main road, which show a positive relationship with vulnerability. Equation (4) is applied to the indicators of GDP per capita and hospital beds per 10,000 population, which show a negative relationship with vulnerability. Each normalized indicator ranges from 0 to 1, where 0 is the lowest contribution to human vulnerability and 1 is the highest contribution to human vulnerability.

Afterwards, the total grid cells of Shanghai are used for cluster analysis, which is performed in the five-dimensional data space spanned by indicators. In this paper, the clustering technique used is an improved *k*-means clustering that uses GA-generated initial cluster centers.

*k*-means clustering aims to search for the solution of partitioning a data set into *k* clusters. Data objects in the same cluster are similar to each other, and objects from distinct clusters are different from each other. This distribution minimizes the SSE of each data object from its cluster center. SSE is a commonly used criterion in measuring the quality of clustering. A lower SSE indicates better partition quality for partitions with the same *k*. This criterion is defined as follows:

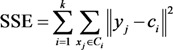
(5)
where *y_j_* is the *j*th object in cluster *C_i_*, and *c_i_* is the center of cluster *C_i_*.

*k*-means clustering algorithm uses *k*-seed objects as initial *k* centers. This clustering algorithm consists of three basic operations performed iteratively, namely, data assignment to a cluster, centers (cluster mean vector) computation, and SSE convergence test. However, different initial centers may lead to different final cluster centers because this clustering algorithm converges to a local minimum. In this study, GA is used to obtain the initial centers for *k*-means clustering to identify reliably and efficiently high quality clustering solutions on the basis of the SSE criterion. The derivative-free optimization strategy, as a type of population-based evolutionary algorithm, allows GA to always yield a global optimum of the objective function.

**Figure 2:**
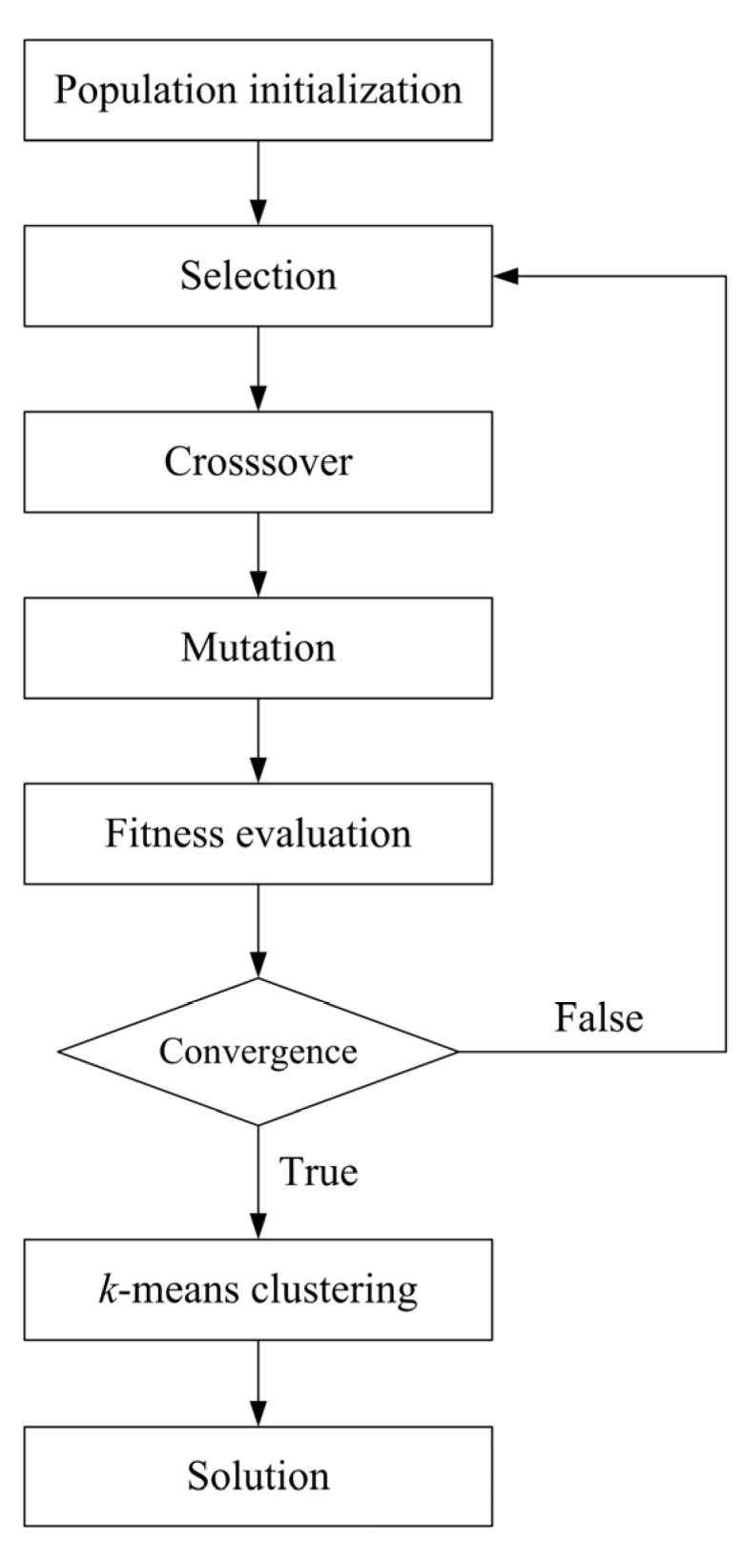
Over flowchart of genetic *k*-means clustering.

The overall procedure of genetic *k*-means clustering algorithm is shown in Figure 2. The algorithm begins with the random initialization of a population and the calculation of the fitness values of the population. Each chromosome in the population denotes a set of *k* cluster centers that use a real-number representation. The fitness function of the population is as follows:

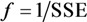
(6)

The GA operators, which consist of selection, crossover, and mutation, are repeatedly conducted. The fitness values of the population are repeatedly evaluated until the fitness function becomes steady in the sense that its value of the best population does not change for several generations. In this case, GA is said to be converged. The best population provided by GA convergence will be close to the global minimum of the SSE. This best population is then inputted as the initial centers of the *k*-means clustering, thus obtaining the global optimum clustering solution.

To determine the optimal clustering number, we introduce the silhouette coefficient to work in combination with the SSE criterion because the SSE criterion is sensitive to the number of clusters, *k*. The silhouette coefficient, a popular method of measuring the clustering quality, which combines both cohesion and separation [33], is rather independent from the number of clusters, *k*. For object *i*, the silhouette coefficient is expressed as follows:

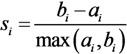
(7)
where *a_i_* is the average distance of object *i* to all other objects in its cluster; for object *i* and any cluster not containing it, calculate the average distance of the object to all the objects in the given cluster, and *b_i_* is the minimum of such values with respect to all clusters.

An overall measure of the goodness of clustering can be obtained by calculating the average silhouette coefficient of all objects. For one clustering with *k* categories, the average silhouette coefficient of the cluster is taking the average of the silhouette coefficients of objects belonging to the clusters; that is:

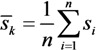
(8)
where *n* is the total number of objects in the data set. The value of the silhouette coefficient can vary between –1 and 1. A higher value indicates better clustering quality.

In the study, we conduct genetic *k*-means clustering analysis of the study area under different *k* values. We plot the curves of the SSE and average silhouette coefficient against the number of clusters to analyze the two curves and to identify the optimal number of clusters, *k*_opt_. The clustering result with *k*_opt_ as the number of clusters is then used to categorize the human vulnerability of the study area. We place the clustering result into space by using GIS to obtain the vulnerability mapping result.

### 2.4. Kruskal-Wallis One-Way ANOVA

To further analyze the validity of the clustering results, Kruskal-Wallis one-way ANOVA is performed to determine whether the clusters are actually different in vulnerability characteristics. Each vulnerability indicator is respectively tested whether its variance is partitioned by the different clusters identified. Summary statistics and box plots are presented to reveal the test results and explore the vulnerability characteristics of clusters.

### 2.5. Information Entropy Analysis and Vulnerability Evaluation

The concept of entropy was first introduced into the information theory by Shannon [34]. In information theory, entropy is a measure of the disorder degree of a system. The larger values of entropy indicate more randomness and thus less information is expressed by data. It can measure the extent of useful information with data provided. Therefore, entropy is an objective means of defining the weights of vulnerability indicators based on the useful information in the available data.

For the study area, the ratio of value of the indicator *j* in grid *i* is defined as:

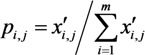
(9)
where *x′_i,j_* is the normalized values of the indicator *j* of the grid *i*; *m* is the total number of the grids in the study area.

Then, the information entropy of the indicator *j* is expressed as:

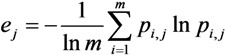
(10)

Therefore, the importance of indicator *j* extracted from the data set is calculated by:

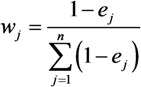
(11)
where *n* is the number of the indicators.

We evaluate the vulnerability of each cluster of the study area by a weighted sum model of the indicators, using the importance of the indicator calculated by Equation (11) as the weight; that is:

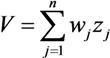
(12)
where *z_j_* is the mean value of the indicator *j* of a cluster.

## 3. Results and Discussion

### 3.1. Spatial Distribution of the Vulnerability Indicators

**Figure 3:**
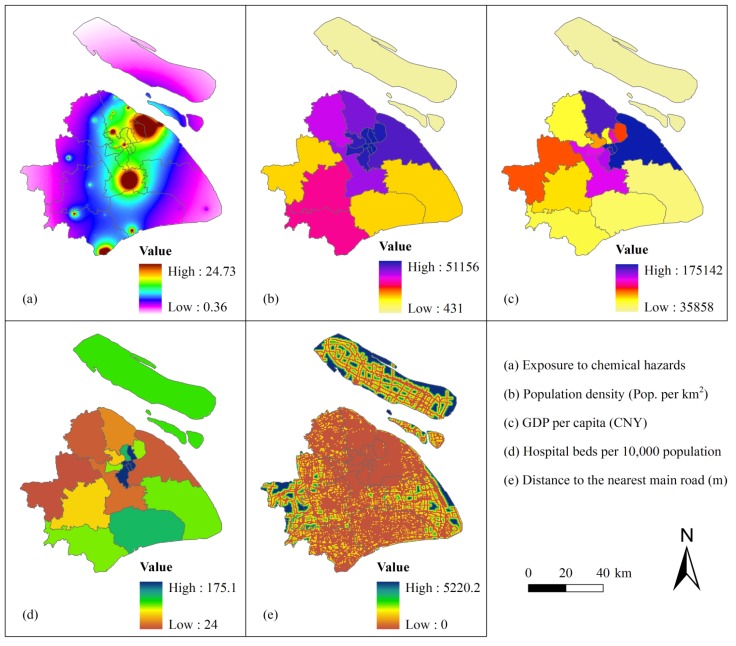
Spatial distribution of indicators of human vulnerability to chemical hazards in Shanghai (CNY: Chinese yuan, the currency of China).

Figure 3 displays the five vulnerability indicators in their spatial distribution. This figure illustrates the location-based action of each indicator that influences vulnerability. Exposure presents the degree of potential threat of chemical hazard to humans. Here, exposure distribution (Figure 3(a)) shows high exposure in the north of Pudong District, center of Minghang District, and south of Jinshan District. High exposure is caused by the numerous corporations and plants gathered in these areas, which increases the risks in these areas.

Population density provides information on the spatial distribution and number of potentially endangered people within the urban. Population distribution (Figure 3(b)) shows high density in the center of Shanghai City, such as Putuo District, Changning District, and Xuhui District, suggesting high sensitivity in these areas.

GDP per capita presents the socioeconomic development level on the spatial distribution in the city. Pudong District has the highest GDP per capita, followed by Baoshan District. Chongming County has the lowest GDP per capita (Figure 3(c)).

Hospital beds per 10,000 population represents the health care service on the spatial distribution in the city. Like most cities, the centers of Shanghai, which include Huangpu District and Xuhui District (Figure 3(d)), have the highest value of this indicator.

Distance to the nearest main road enables a geospatial assessment of accessibility to social resources. The road networks are shown to be well developed in the center of the city (Figure 3(e)). However, the road networks are undeveloped in several places far from the city center, particularly in Chongming County. The indicators GDP per capital, hospital beds per 10,000 population, and distance to the nearest main road enable identification of the coping capacity of an area during and after emergencies.

### 3.2. Vulnerability Mapping

We perform genetic *k*-means clustering analysis with normalized vulnerability indicators of the grid cells in the study area. The clustering number should be less than 15 because of the moderate size of the study area. To determine the optimal clustering number, we conduct clustering analysis on 2 clusters to 15 clusters and calculate the SSE and average silhouette coefficient *versus* the clustering number.

**Figure 4:**
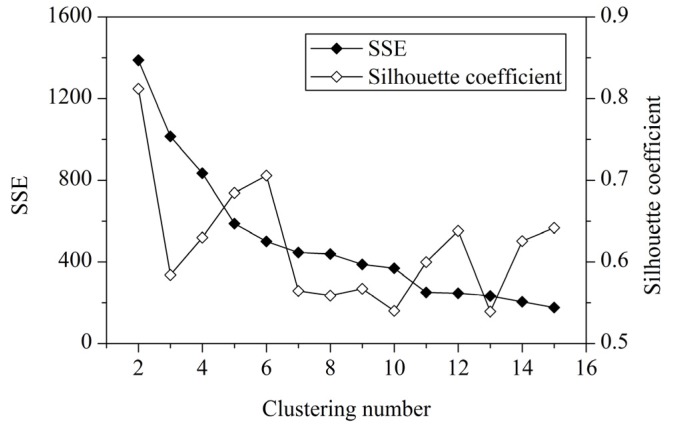
SSE and average silhouette coefficient *versus* number of clusters.

The optimal clustering number can be found in a data set by looking for the number of clusters at which a knee, peak, or dip exists in the plot of the evaluation measure when plotted against the number of clusters [33]. Figure 4 shows the plots of the SSE and average silhouette coefficient *versus* the number of clusters for the genetic *k*-means clustering of the study area. A distinct knee in the SSE and a distinct peak in the silhouette coefficient are present when the number of clusters is equal to 6.

**Figure 5:**
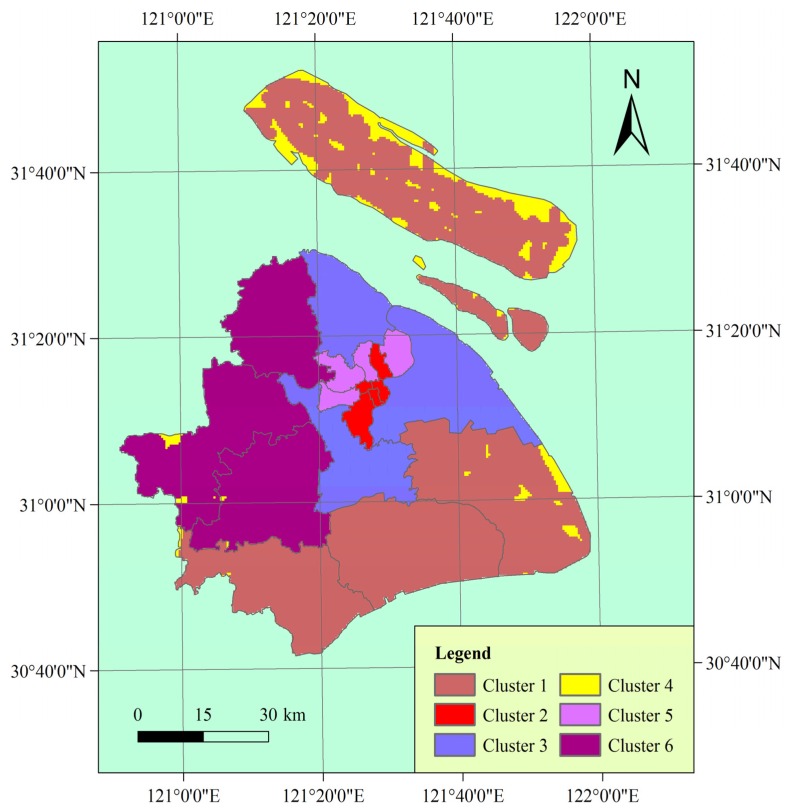
Vulnerability mapping of the study area.

Generally, a higher average silhouette coefficient indicates better clustering quality. In this view, the optimal clustering number of grid cells in the study area should be 2, at which the value of the average silhouette coefficient is highest. However, the SSE of this clustering solution (*k* = 2) is too large. At *k* = 6, the SSE is much lower. In addition, the value of the average silhouette coefficient at *k* = 6 is also very high, which is just lower than *k* = 2. Thus, we use the clustering result at *k* = 6 for vulnerability mapping. Thereafter, the spatial distribution of vulnerability mapping is shown in Figure 5.

**Figure 6:**
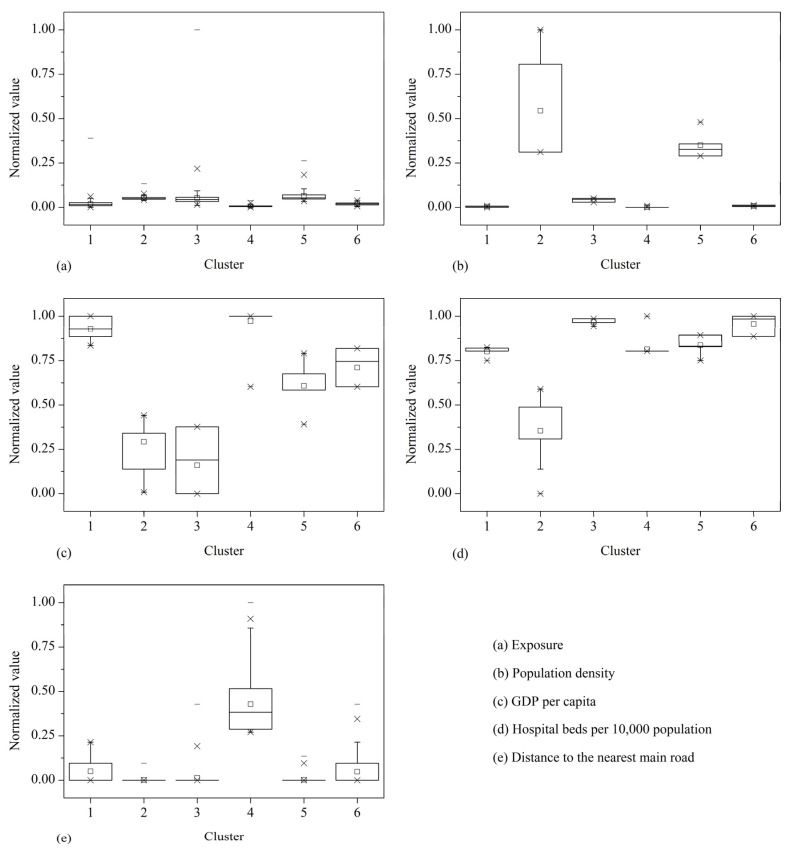
Box plots of vulnerability indicators against clusters.

The results of Kruskal-Wallis one-way ANOVA of each vulnerability indicator with respect to six clusters are shown in Table 1. It shows that all the vulnerability indicators exhibit significant (*P* < 0.01) variation in the 6 clusters, which demonstrate that the vulnerability characteristics of the study area are effectively categorized. The box plots of the vulnerability indicators are show in Figure 6.

**Table 1:** Kruskal-Wallis one-way ANOVA of vulnerability indicators by six clusters.

Vulnerability indicators	Chi-Square	DF	Prob > Chi-Square
Exposure	12,522.2	5	0
Population density	16,870.7	5	0
GDP per capita	23,382.7	5	0
Hospital beds per 10,000 population	21,105.7	5	0
Distance to the nearest main road	7,058.82	5	0

As shown in Figure 6, the combinations of vulnerability indicators represent typical patterns of human vulnerability to chemical hazards. This allows easy discernment of how the six clusters differ in each dimension. Clusters 1 and 4 have the lowest population density and the lowest GDP per capita in the study area. Note that for GDP per capita and hospital beds per 10,000 population, a larger normalized value indicates smaller observed value because of the negative relationship of these two indicators with vulnerability. GDP per capita can reflect the socioeconomic development level of an area, which is in correlation with the high value of distance to the nearest main road in these two clusters. In particular, Cluster 4 has the highest value of distance to the nearest main road in the study area. The hospital beds per 10,000 population in Cluster 4 is not the lowest because of the low population density of the cluster. Therefore, these two clusters represent low coping capacity in the study area. Because of their low ability to respond to chemical hazard, people in these two clusters will be potentially in high danger although the exposure of these two clusters is low.

Clusters 2, 3, and 5 show the highest exposure and the highest population density in the study area (Figure 3(a), Figure 5, Figure 6). In particular, the population density of Clusters 2 and 5 are much higher compared with other clusters. These two clusters are potentially in the highest danger on the basis of exposure and population density. Nevertheless, the infrastructure of Clusters 2 and 5 is relatively well because these clusters are located in the center of the city, resulting in high coping capacity. Among all the clusters, Clusters 2 and 5 have the most developed road networks, as indicated by the proximity of these clusters to the nearest main road (Figure 3(e) , Figure 6(e)). Moreover, the hospital beds per 10,000 population of these two clusters are relatively high although these clusters have the highest population density. Additionally, the hospital beds per 10,000 population of Cluster 2 is the highest in the study area. Cluster 3 has the highest GDP per capita among all the clusters.

The exposure, population density, and GDP per capita of Cluster 6 are moderate. Cluster 6 has the second lowest hospital beds per 10,000 population, very close to that of Cluster 3, which has the lowest hospital beds per 10,000 population. The road networks of Cluster 6 are also not well developed.

The identified vulnerability clusters describe typical indicator combinations, with the specific characteristics of vulnerability. Thus, each cluster shows specific causes of vulnerability and opportunities to increase the ability to cope with hazard.

### 3.3. Human Vulnerability Evaluation and Reduction Points

**Figure 7:**
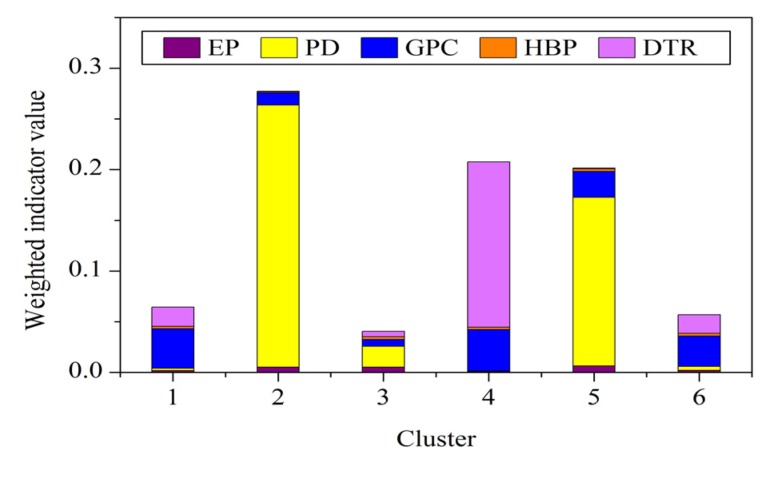
Weighted indicator value stack column plot of clusters (EP: exposure; PD: population density; GPC: GDP per capita; HBP: hospital beds per 10,000 population; DTR: distance to the nearest main road).

According to Equations (10) and (11), information entropies and weights of vulnerability indicators are listed in Table 2. The indicators of each cluster are piled by using weighted sum according to Equation (12), which is characterized by a column as depicted in Figure 7. The total height of columns corresponds to the vulnerability evaluation results of the clusters.

**Table 2:** Information entropies and weights of vulnerability indicators.

Vulnerability indicators	Infromation entropy ( *e_j_*)	Weight ( *w_j_*)
Exposure	0.9684	0.0997
Population density	0.8495	0.4746
GDP per capita	0.9866	0.0421
Hospital beds per 10,000 population	0.9991	0.0028
Distance to the nearest main road	0.8793	0.3807

Cluster 2 represents the most vulnerable regions according to evaluation results (Figure 7), which is mainly contributed by the high population density. Clusters 4 and 5 display the close vulnerability values. However, the vulnerability characteristics of these two clusters are significantly different. Cluster 4 is mainly contributed by the long distance to the nearest main road, whereas Cluster 5 is mainly contributed by the high population density. Clusters 1 and 6 also display close vulnerability values. Indicated by Figure 6, Figure 7, it can be found that Cluster 1 has lower GDP per capita and more hospital beds per 10,000 population compared with Cluster 6. These demonstrate that areas exhibiting similar evaluation results may have distinct vulnerability dimensions, which we maintained at the beginning of the study.

The variation between vulnerability dimensions in the study area suggests that no single option for reducing vulnerability is suitable for all the area. However, vulnerability mapping through clustering enables the tailoring of appropriate vulnerability reducing points to apply in different clusters. The vulnerability indicators are similar within the same cluster but distinct with the other clusters, thereby effectively applying specific vulnerability-reducing interventions.

Clusters 1 and 4, particularly cluster 4, would require high attention in infrastructure development, such as improvement of road networks and social service enhancement. This development will increase the ability of people to access social resources, thus improving the ability of people to cope with chemical hazards. In addition, more attention should be paid to socioeconomic development. The GDP per capita usually reflects the available resources of people to cope with hazards and to support social services and high-quality infrastructure. When the coping capacity of these clusters is enhanced, the human vulnerability of these two clusters will become significantly low as the exposure to chemical hazard is low.

The areas of Clusters 2 and 5 are in the center of the city, which has the highest population density. Although the infrastructure of the areas are well developed and the medical care level of Cluster 2 is the best in the study area, automatic monitoring and alarm systems should be set up in these areas to reduce human vulnerability as the exposure of these two clusters is high. In addition, a detailed evacuation plan should be constituted to ensure a systematic evacuation and transfer of people in case of chemical hazard.

Several special measures should be considered to cope with high exposure in Cluster 3. The medical care level of Cluster 3 is insufficient for the population requirement. Apart from enhancing the medical care level of this cluster, a professional succor group, which rescues and transfers people injured by chemical hazard, should be constituted.

The medical care level and infrastructure, especially the medical care level, in Cluster 6 need to improve. Several open spaces are observed in Cluster 6 given its low population density. Therefore, establishing refuges is a better option to protect people from the effects of moderate exposure. With the combination of the aforementioned measures, the human vulnerability of this area would be reduced.

## 4. Conclusions

The vulnerability mapping presented in this paper outlines one way of dealing with vulnerability-reducing interventions in an industrialized city. The clustering approach enables the discernment of vulnerability dimensions. The toxicity of each hazardous substance during exposure is considered to characterize the human vulnerability to chemical hazard of the study area. Therefore, proximity depiction of exposure becomes more accurate through this approach. Sensitivity mainly considers the spatial concentration and distribution of people, whereas coping capacity mainly considers the socioeconomic development level and medical care level of an area.

Vulnerability mapping through clustering analysis identifies similar characteristics of vulnerability within one cluster as well as the differences of one cluster with other clusters. Thus, vulnerability mapping facilitates the respective vulnerability-reducing policy applications. *k*-means clustering improved by GA ensures the global optimal clustering solution on the defined *k* cluster number. The SSE and average silhouette coefficient evaluations enable us to measure the quality of clustering and to determine the optimal cluster number of vulnerability mapping.

The human vulnerability of the study area is mapped with six clusters. The results reflect the combinations of cluster-specific vulnerability dimensions and spatial distribution. According to the specific characteristics of vulnerability in each cluster, we suggest the specific points for vulnerability reduction to stimulate new insights on reducing human vulnerability and to respond to the need of rationally allocating the limited funds available for vulnerability reduction.

## References

[1] Malich G., Braun M., Loullis P., Winder C. (1998). Comparison of regulations concerning hazardous substances from an international perspective. J. Hazard. Mater..

[2] Adger W.N. (2006). Vulnerability. Glob. Environ. Change.

[3] Adger W.N., Agrawala S., Mirza M.M.Q., Conde C., O’Brien K., Pulhin J., Pulwarty R., Smit B., Takahashi K., Parry M.L., Canziani O.F., Palutikof J.P., van der Linden P.J., Hanson C.E. (2007). Assessment of adaptation practices, options, constraints and capacity. Climate Change 2007: Impacts, Adaptation and Vulnerability.

[4] Kelly P.M., Adger W.N. (2000). Theory and practice in assessing vulnerability to climate change and facilitating adaptation. Clim. Change.

[5] Luers A.L., Lobell D.B., Sklar L.S., Addams C.L., Matson P.A. (2003). A method for quantifying vulnerability, applied to the agricultural system of the Yaqui Valley, Mexico. Glob. Environ. Change.

[6] Preston B.L., Brooke C., Measham T.G., Smith T., Gorddard R. (2009). Igniting change in local government: Lessons learned from a bushfire vulnerability assessment. Mitig. Adapt. Strateg. Glob. Change.

[7] Birkmann J., Birkmann J. (2006). Measuring vulnerability to promote disaster-resilient societies: Conceptual frameworks and definitions. Measuring Vulnerability to Natural Hazards-Towards Disaster Resilient Societies.

[8] Janssen M.A., Schoon M.L., Ke W., Börner K. (2006). Scholarly networks on resilience, vulnerability and adaptation within the human dimensions of global environmental change. Glob. Environ. Change.

[9] Füssel H.M. (2007). Vulnerability, a generally applicable conceptual framework for climate change research. Glob. Environ. Change.

[10] Bone C., Alessa L., Altaweel M., Kliskey A., Lammers R. (2011). Assessing the impacts of local knowledge and technology on climate change vulnerability in remote communities. Int. J. Environ. Res. Public Health.

[11] Kim E.S., Choi H.I. (2011). Assessment of vulnerability to extreme flash floods in design storms. Int. J. Environ. Res. Public Health.

[12] Huang G.L., London J.K. (2012). Cumulative environmental vulnerability and environmental justice in California’s San Joaquin Valley. Int. J. Environ. Res. Public Health.

[13] Liverman D., Kasperson J.X., Kasperson R.E. (2001). Vulnerability to global environmental change. Global Environmental Risk.

[14] Malone E.L., Engle N.L. (2011). Evaluating regional vulnerability to climate change: Purposes and methods. Rev. Clim. Change.

[15] Turner B.L., Kasperson R.E., Matson P.A., McCarthy J.J., Corell R.W., Christensen L., Eckley N., Kasperson J.X., Luers A., Martello M.L. (2003). A framework for vulnerability analysis in sustainability science. Proc. Natl. Acad. Sci. USA.

[16] Schneider S., Sarukhan J., McCarthy J.J., Canziani O.F., Leary N.A., Dokken D.J., White K.S. (2001). Overview of impacts, adaptation, and vulnerability to climate change. Climate Change 2001: Impacts, Adaptation, and Vulnerability.

[17] Turner B.L., Matson P.A., McCarthy J.J., Corell R.W., Christensen L., Eckley N., Hovelsrud-Broda G.K., Kasperson J.X., Kasperson R.E., Luers A. (2003). Illustrating the coupled human-environment system for vulnerability analysis: Three case studies. Proc. Natl. Acad. Sci. USA.

[18] Kasperson R.E., Dow K., Archer E.R.M., Cáceres D., Downing T.E., Elmqvist T., Eriksen S., Folke C., Han G., Iyengar K., Hassan R., Scholes R., Ash N. (2005). Vulnerable people and places. Ecosystems and Human Well-Being: Current State and Trends.

[19] Smit B., Wandel J. (2006). Adaptation, adaptive capacity and vulnerability. Glob. Environ. Change.

[20] Clark G.E., Moser S.C., Ratick S.J., Dow K., Meyer W.B., Emani S., Weigen J., Kasperson J.X., Kasperson R.E., Schwarz H.E. (1998). Assessing the vulnerability of coastal communities to extreme storms: The case of Revere, MA, USA. Mitig. Adapt. Strateg. Glob. Change.

[21] Preston B.L., Yuen E.J., Westaway R.M. (2011). Putting vulnerability to climate change on the map: A review of approaches, benefits, and risks. Sustain. Sci..

[22] de la Torre A., Iglesias I., Carballo M., Ramirez P., Munoz M.J. (2012). An approach for mapping the vulnerability of European Union soils to antibiotic contamination. Sci. Total. Environ..

[23] Petschel-Held G., Block A., Cassel-Gintz M., Kropp J., Lüdeke M.K.B., Moldenhauer O., Reusswig F., Schellnhuber H.J. (1999). Syndromes of global change: A qualitative modelling approach to assist global environmental management. Environ. Modell. Assess..

[24] O’Brien K.L., Leichenko R., Kelkar U., Venemad H., Aandahl G., Tompkins H., Javed A., Bhadwal S., Barg S., Nygaard L., West J. (2004). Mapping vulnerability to multiple stressors: Climate change and globalization in India. Glob. Environ. Change.

[25] Yan L., Xu X.G. (2010). Assessing the vulnerability of social-environmental system from the perspective of hazard, sensitivity, and resilience: A case study of Beijing, China. Environ. Earth Sci..

[26] Chang C.L., Chao Y.C. (2012). Using the analytical hierarchy process to assess the environmental vulnerabilities of basins in Taiwan. Environ. Monit. Assess..

[27] Zou B., Wilson J.G., Zhan F.B., Zeng Y.N. (2009). An emission-weighted proximity model for air pollution exposure assessment. Sci. Total. Environ..

[28] Bross I. (1950). Estimates of the LD_50_: A critique. Biometrics.

[29] Weil C.S. (1952). Tables for convenient calculation of median-effective dose (LD_50_ or ED_50_) and instructions in their use. Biometrics.

[30] Petrick J.S., Jagadish B., Mash E.A., Aposhian H.V. (2001). Monomethylarsonous acid (MMA(III)) and arsenite: LD_50_ in hamsters and *in vitro* inhibition of pyruvate dehydrogenase. Chem. Res. Toxicol..

[31] Zbinden G., Fluryroversi M. (1981). Significance of the LD_50_-test for the toxicological evaluation of chemical-substances. Arch. Toxicol..

[32] Schlede E., Mischke U., Roll R., Kayser D. (1992). A national validation-study of the acute-toxic-class method—An alternative to the LD50 test. Arch. Toxicol..

[33] Tan P.N., Steinbach M., Kumar V. (2006). Introduction to Data Mining.

[34] Shannon C.E. (1948). A mathematical theory of communication. Bell Syst. Tech. J..

